# Supplementary material for: A Critical View of the Application of the APEX Software (Aqueous Photochemistry of Environmentally-Occurring Xenobiotics) to Predict Photoreaction Kinetics in Surface Freshwaters
Source: Molecules. 2019 Dec 18;25(1):9. doi: 10.3390/molecules25010009 (PMC7017383; doi:10.3390/molecules25010009)
Supplement: Supplementary file 1 [file molecules-25-00009-s001.zip › 3.2.4_gcc-4.4.0.zip!/3.2.4_gcc-4.4.0/3.2.4_gcc-4.4.0/doc/octave/html/interpreter/Notes-for-the-C-programmer.html]

Notes for the C programmer - Untitled


Up: The <code>switch</code> Statement


---

#### 10.2.1 Notes for the C programmer

The `switch` statement is also available in the widely used C
programming language. There are, however, some differences
between the statement in Octave and C

- Cases are exclusive, so they don't `fall through' as do the cases
  in the `switch` statement of the C language.- The command\_list elements are not optional. Making the list
    optional would have meant requiring a separator between the label and
    the command list. Otherwise, things like

    ```
              switch (foo)
                case (1) -2
                ...
    ```

    would produce surprising results, as would

    ```
              switch (foo)
                case (1)
                case (2)
                  doit ();
                ...
    ```

    particularly for C programmers. If `doit()` should be executed if
    foo is either `1` or `2`, the above code should be
    written with a cell array like this

    ```
              switch (foo)
                case { 1, 2 }
                  doit ();
                ...
    ```
